# Transcriptome Analysis Elucidates the Potential Key Genes Involved in Rib Development in *bmp6*-Deficient Silver Carp (*Hypophthalmichthys molitrix*)

**DOI:** 10.3390/ani14101451

**Published:** 2024-05-13

**Authors:** Xiaohui Li, Chunyan Zhang, Cui Feng, Zewen Zhang, Nannan Feng, Hang Sha, Xiangzhong Luo, Guiwei Zou, Hongwei Liang

**Affiliations:** 1 Yangtze River Fisheries Research Institude, Chinese Academy of Fishery Sciences, Wuhan 430223, China; lixiaohui@yfi.ac.cn (X.L.); zhangchunyan@yfi.ac.cn (C.Z.); fengcui@yfi.ac.cn (C.F.); zhangzewen@yfi.ac.cn (Z.Z.); fengnannan@yfi.ac.cn (N.F.); sh1812@yfi.ac.cn (H.S.); lxz@yfi.ac.cn (X.L.); zougw@yfi.ac.cn (G.Z.); 2 Laboratory of Zooligical Systematics and Application of Hebei Province, College of Life Sciences, Hebei University, Baoding 071002, China; 3 Hubei Hongshan Laboratory, Huazhong Agricultural University, Wuhan 430070, China

**Keywords:** *bmp6*, rib, *Hypophthalmichthys molitrix*, transcriptome analysis

## Abstract

**Simple Summary:**

Bone morphogenetic protein 6 (BMP-6) is known for its ability to stimulate bone and cartilage formation. The rib cage is an important skeletal structure; however, there has been limited research undertaken on the effects of the *bmp6* gene on rib development. This study showcases the effective utilisation of CRISPR/Cas9 technology for the purpose of disrupting the *bmp6* gene in silver carp, leading to the creation of chimeras in the P_0_ generation, marking the first instance of such an achievement. The chimeras exhibited complete viability, normal appearance, and partial intermuscular bones loss, with approximately 30% of them displaying rib bifurcation or bending. Subsequently, a transcriptome analysis on ribs of P_0_ chimeras and wild-type silver carp was conducted, leading to the identification of 934 genes exhibiting differential expression. A KEGG analysis revealed that the up-regulated genes such as *tnfα*, *fos*, and *ctgf* may facilitate the proliferation and differentiation of osteoclasts, whereas the down-regulation of genes such as *tgfb2* and *tgfbr1* may hinder the formation and specialisation of osteoblasts, ultimately resulting in rib abnormalities. This study presents novel findings on the impact of *bmp6* gene deletion on the rib development of silver carp, while simultaneously investigating the previously unexplored molecular mechanisms underlying rib defects in fish.

**Abstract:**

Bone morphogenetic protein 6 (BMP-6) is a constituent of the TGF-β superfamily, known for its ability to stimulate bone and cartilage formation. The investigation of *bmp6*’s involvement in the formation of intermuscular bones in fish has garnered significant attention in recent years. The rib cage is an important skeletal structure that plays a protective function for internal organs in fish. However, there has been limited research conducted on the effects of the bmp6 gene on rib development. Silver carp is one of four major fish in China, favoured for its affordability and tender muscle. Nevertheless, the presence of numerous intermuscular bones in silver carp significantly hinders the advancement of its palatability and suitability for processing. This study showcases the effective utilisation of CRISPR/Cas9 technology for the purpose of disrupting the *bmp6* gene in silver carp, leading to the creation of chimeras in the P_0_ generation, marking the first instance of such an achievement. The chimeras exhibited complete viability, normal appearance, and partial intermuscular bones loss, with approximately 30% of them displaying rib bifurcation or bending. Subsequently, a transcriptome analysis on ribs of P_0_ chimeras and wild-type silver carp was conducted, leading to the identification of 934 genes exhibiting differential expression, of which 483 were found to be up-regulated and 451 were found to be down-regulated. The results of the KEGG analysis revealed that the “NF-kappa B signalling pathway”, “Hippo signalling pathway”, “osteoclast differentiation”, and “haematopoietic cell lineage” exhibited enrichment and displayed a significant correlation with bone development. The up-regulated genes such as *tnfα*, *fos*, and *ctgf* in pathways may facilitate the proliferation and differentiation of osteoclasts, whereas the down-regulation of genes such as *tgfb2* and *tgfbr1* in pathways may hinder the formation and specialisation of osteoblasts, ultimately resulting in rib abnormalities. This study presents novel findings on the impact of bmp6 gene deletion on the rib development of silver carp, while simultaneously investigating the previously unexplored molecular mechanisms underlying rib defects in fish.

## 1. Introduction

Bone morphogenetic proteins (BMPs) are members of the transforming growth factor-β (TGF-β) superfamily [1,2]. These proteins transmit signals to target cells via the BMP type I (ACVR1A (Alk2), BMPR1A (Alk3), and BMPR1B (Alk6)) and type II (BMPR2, ActRIIA, and ActRIIB) serine/threonine kinase receptor heterooligomeric complexes, thereby influencing cellular responses [3]. Despite BMPs exhibiting diverse functions in the development of various organ and tissue systems, they were initial identified as inducers of bone and cartilage formation [4]. Mutations induced in bmp6 were discovered to play significant roles in survival, growth, and tooth growth [5]. In murine models, *bmp6* was identified as a direct target of miR-451a, which modulates bone formation by regulating the expression of SMAD1/5/8. Decreasing the levels of miR-451a expression could potentially enhance bone formation during the progression of osteoporosis [6]. The knockdown of bmp6 in zebrafish leads to the up-regulation of sik1 and the activation of TNF-α signalling through the NF-κB pathway, resulting in the inhibition of osteoblast development and the promotion of osteoclast formation. Consequently, this inhibits the formation of intermuscular bones (IBs) [7]. Furthermore, it was reported that a new strain of Carassius auratus without IBs was generated by knocking down bmp6 [8].

In teleost, the rib is an integral component of the axial skeleton, extending ventrally and laterally from the vertebrae to the subperitoneal fascia on both sides of the abdominal cavity [9,10]. The rib is anatomically linked to collagen-rich tissue and Sharpey’s fibres [11]. Research conducted on fish has demonstrated that the rib undergoes development via perichondral ossification and elongation through type II endochondral ossification, without the formation of bone tissue within the medullary cavity [12]. It is well known that fish do not have bone marrow tissue because haematopoiesis takes place mainly in the kidneys and the internal cavity of the rib is almost occupied by chondrocyte-like cells [13]. Upon examination of the bone’s microstructure using SEM, it becomes evident that all fibres within the rib tissue exhibit a pronounced alignment along the bone’s longitudinal axis [14]. This particular orientation of collagen fibres plays a crucial role in maintaining the rib’s strength [15]. Additionally, the ribs, in conjunction with the robust abdominal muscle tissue, are enveloped by the peritoneal wall, which serves as a protective barrier for the abdominal organs. They also act as anchors for muscle attachment, thus contributing to the swimming movement of the fish. A recent study found that the knockout of the *runx2b* gene in amphitriploid gibel carp led to a reduction in or absence of IBs, with half of the IB-free individuals exhibiting subtle defects in the ribs such as bifurcation and warping [16]. Similarly to *runx2b*, as a key gene affecting the occurrence of intermuscular bones, the role of the *bmp6* gene in the development of fish ribs is still unknown.

Silver carp (Hypophthalmichthys molitrix) is one of the four major carp species cultivated in China [17], and its aquaculture achieved a total production of 3,879,800 tons in 2022, accompanied by an annual output value exceeding 30 billion yuan, thereby signifying the industry’s significant prominence. Silver carp feed on phytoplankton without additional feeding, so aquaculture of silver carp reduces feeding costs [18]. Today, silver carp have been introduced or have spread to many countries worldwide [19], not only to increase breeding benefits, but also to reduce algal blooms and improve water quality [20,21,22]. However, silver carp exhibit a higher abundance of IBs, with a significant portion of these spines being diminutive in size and possessing intricate branching structures, thereby exerting a substantial detrimental impact on both the palatability and suitability for processing.

Due to the long reproduction cycle, there are no gene-edited silver carp strains so far. In this study, we knocked out the *bmp6* gene on silver carp by CRISPR/Cas9 technology for the first time, leading to the creation of the chimeras in the P_0_ generation, which were fully viable and appeared normal. The chimeras were subjected to alizarin red staining, revealing that approximately 70% of them exhibited partial IBs loss, while approximately 30% displayed rib bifurcation or bending when compared to the wild-type silver carp. To investigate the role of bmp6 in rib development, a transcriptome analysis of the ribs of P_0_ chimeras and wild-type silver carp was conducted, respectively. The results will be valuable for the study of molecular mechanisms of rib development in silver carp and provide a useful tool for the application of gene editing technology in silver carp molecular breeding.

## 2. Materials and Methods

### 2.1. Experimental Fish and Maintenance

The research subjects utilised in this experiment were obtained from the Genetics and Breeding Center of Silver Carp, Ministry of Agriculture and Rural Affairs in Jingzhou city, Hubei province, China. The parental organisms were raised in 500 m^2^ ponds and administered with an oxytocin drug to facilitate successful silver carp spawning. Among the four large fish species, chub typically exhibits earlier maturation and is the first to spawn. In the middle and lower reaches of the Yangtze River, the optimal spawning season occurs from mid-May to mid-June. Three types of oxytocins are used for inducing spawning: pituitary extract, chorionic gonadotropin, and LRH-A analogue. The recommended dosage for inducing spawning is either 2 mg of pituitary extract combined with 800–1200 IU/kg of hormone, or 0.5–1 mg of pituitary extract combined with 10–30 micrograms/kg of LRH-A analogue in a single injection. Following hormone administration, the parent fish will enter a state of heat and subsequently spawn autonomously after a certain period of time. In our study, the fertilised eggs were then incubated at a temperature range of 22 °C to 23 °C. Following hatching, the larvae were fed with brine shrimp on a three-times-daily basis until they attained a body length of 2 cm to 3 cm in 0.1 m^3^ tank. Subsequently, the larvae were transferred to a 16 m^2^ algae-enriched pond.

### 2.2. Bmp6 Gene Editing by CRISPR/Cas9

The *bmp6* gene sequence was obtained from the silver carp genome (unpublished). The CRISPRscan website (https://www.crisprscan.org/) (accessed on 10 February 2024) was used to design guide RNA (gRNA) targets (Table S1), and the gRNA was synthesised using the NEB HiScribe T7 Rapid and Efficient RNA Synthesis Kit (NEB E2050S, USA) (Wuhan, China). Amounts of 400–600 pg of sgRNA and 0.2–0.4 pmol of Cas9 NLS nuclease (NEB M0646T, USA) were co-injected into the single-cell eggs of silver carp.

### 2.3. Mutant Fish Identification

The injected eggs were raised to 6 months old, and the genomic DNA was extracted from fin clips using the simplified alkaline-lysis method. The fin clips were subjected to lysis by adding 100 μL of 50 mM NaOH solution, and then incubating at 94 °C for 50 min, followed by incubation at 4 °C for 10 min. After sufficient vortexing, 10 µL of 1 M Tris-HCl (pH 8.0) was added as the neutralizer. Subsequently, the lysates were centrifuged and the supernatant was used for PCR amplification with primers showed in Table S1. PCR was performed using a 50 μL volume consisting of 1 μL of genomic DNA supernatant, 1 μL of 10 μM forward and reverse primers, 25 μL of 2× Dream Taq Master Mix (Thermo Fisher, Wuhan, China), and 22 μL of H_2_O. The PCR amplification procedure was as follows: 95 °C for 3 min, followed by 34 cycles of denaturation at 95 °C for 30 s, annealing at 60 °C for 30 s, and extension at 72 °C for 30 s, with a final extension step at 72 °C for 2 min. The PCR products were purified and sent to Tianyihuiyuan Biotechnology Company (Wuhan, China) for sequence validation using Sanger sequencing.

Prior to experimental group sampling, the caudal fins of the fish were clipped and sequenced to ensure the usage of successful knockout animals. Fish presenting curved or bifurcated ribs were selected for sampling, triplicates were collected simultaneously. 

### 2.4. Phenotypic Observation

Alizarin red staining was used to observe the skeletal pattern of P_0_ chimeras and wild-type silver carp. The 4-month-old fish were subjected to anaesthesia using a solution of tricaine methanesulphonate (MS-222, Sigma, St. Louis, MO, USA)at a concentration of 200 mg/L before being fixed with 4% paraformaldehyde (PFA) for a duration of 3 days, and alizarin red staining was performed following similar methods to those previously published [16]. Samples were observed using a light microscope (SZM, Shunyu, Ningbo, China), and images were obtained with a charge-coupled device camera (LightTools, Hangzhou, China) coupled to the microscope.

### 2.5. Total RNA Isolation and Sequencing

After anaesthesia with MS-222 (Sigma, USA), ribs were obtained from 6-month-old P_0_ chimeras and wild-type silver carp. Total RNA was isolated using TRIzol Reagent (Invitrogen, Carlsbad, CA, USA), and cDNA library construction and RNA sequencing were performed by Shanghai Bioprofile Biotechnology Co., Ltd. (Shanghai, China). The cDNA libraries were sequenced using the Illumina HiSeq™ 4000 system (Bioprofile, Shanghai, China), which produced 150 bp paired-end reads. The data were available at NCBI SRA database (BioProject ID: PRJNA1092811). All procedures were approved by the Animal Care and Use Committee of the Yangtze River Research Institute, Chinese Academy of Fisheries Sciences (Wuhan, China).

### 2.6. Differentially Expressed Genes Analysis

Differentially expressed genes (DEGs) were selected with |log2 fold change| > 1 and FDR < 0.05 using edgeR. Volcano plots of DEGs were drawn using the R language ggplots2 software package. We used the R-language Pheatmap (1.0.8) software package to perform bi-directional clustering analysis of all different genes of samples. GO enrichment analysis was performed using the topGO package (version 2.38.1). KEGG pathway enrichment analysis was performed using KEGG Mapper (https://www.genome.jp/kegg/tool/map_pathway2.html) (accessed on 10 January 2024) [23].

### 2.7. Quantitative Real-Time PCR (qRT-PCR) Analysis

DEGs were validated by qRT-PCR, and gene-specific primers, as shown in Table S1, were designed using Primer Premier 5 software. qRT-PCR was performed on the Quantstudio 5 Flex PCR system using 2× SYBR Green MasterMix reagent (Vazyme, Nanjing, China) as described previously [23]. Gene expression levels were calculated using the comparative Ct method (2^−ΔΔCt^) [24].

### 2.8. Statistical Analysis

All values are expressed as mean ± SD. Experimental measurements were repeated at least three times. Data were analysed using SPSS v15.0 (IBM Corp., Armonk, NY, USA). The comparisons of gene relative expression level between the WT and CH groups were performed using independent Student *t*-test. *p* < 0.05 was considered statistically significant.

## 3. Results

### 3.1. Bmp6 Gene Editing in Silver Carp

**Figure 1:**
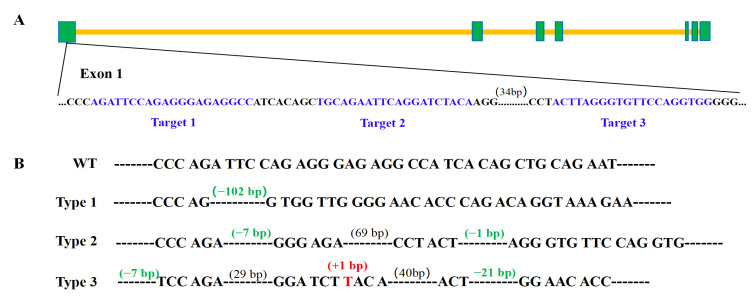
Knocking out *bmp6* gene by CRISPR/Cas9 technology in silver carp. (**A**) Schematic representation of *bmp6* gene structure in silver carp shows the three gRNA targets located within exon 1. The green squares represent exons, the yellow squares represent introns, and the gRNA target sequences are indicated in blue. (**B**) Schematic diagram illustrates three distinct instances of bmp6 mutation types observed in P_0_ chimeras. WT denotes the sequence of the *bmp6* gene in the wild-type silver carp. Type 1–3 signifies the frameshift mutations identified in the *bmp6* gene of the P_0_ chimeras.

In order to examine the involvement of the *bmp6* gene in rib development in silver carp, *bmp6* gene editing in chimeras in the P_0_ generation was achieved using CRISPR/Cas9 technology. Three gRNA target sites were designed in exon1 of bmp6 loci (Figure 1A). Through the utilisation of Sanger sequencing, we detected different types of bmp6 frameshift mutations within every single chimera. Figure 1B displays three instances of the identified mutation types. Type 1 exhibits a 102 bp deletion spanning from the first target site to the third target site, type 2 consists of a 7 bp deletion in the first target site and a 1 bp deletion in the third target site, and type 3 comprises a 7 bp deletion in the first target site, a 1 bp insertion in the second target site, and a 21 bp deletion in the third target site.

### 3.2. Phenotypic Observation in P_0_ Chimeras

**Figure 2:**
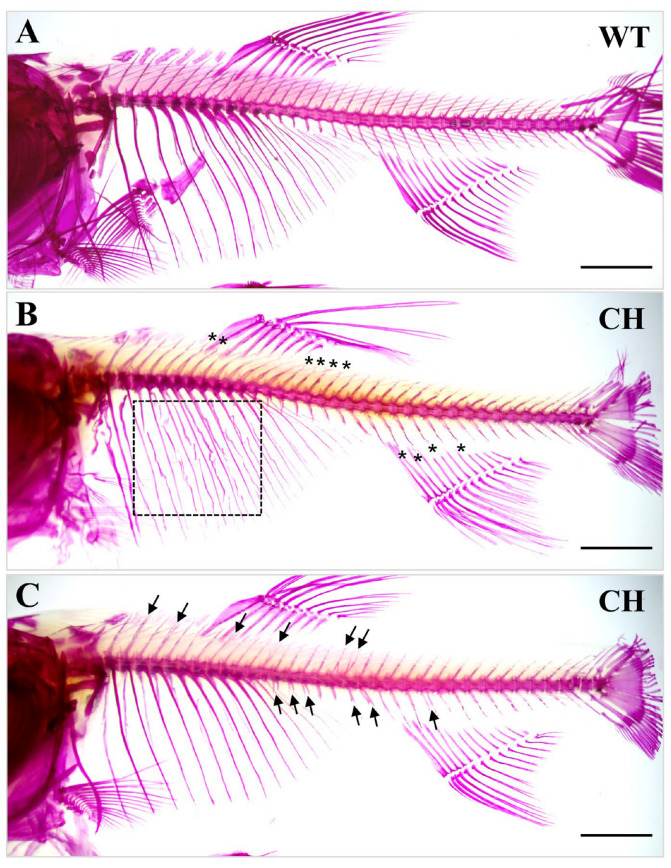
Alizarin Red staining of WT and P0 chimera individuals. Scale bar = 2 mm. Chimeras with a knockout of the *bmp6* gene exhibit curved and bifurcate rib phenotypes, as indicated by dashed lines and asterisks representing curved ribs, and arrows representing bifurcate ribs. (**A**), wild-type silver carp; (**B**), chimeric rib-curved silver carp; (**C**), chimeric rib-bifurcate silver carp. WT: wild-type silver carp; CH: P0 chimeras silver carp.

The combined triple-target knockout of *bmp6* led to a highly efficient of 86.11% in the P_0_ generation (Table 1). Compared to wild-type silver carp, the chimeras are fully viable and appeared normal. Alizarin red staining shows that approximately 70% of the chimeras displays partial loss of intermuscular bones, and with approximately 30% of the chimeras displaying subtle rib bifurcation or bending (Table 1, Figure 2).

### 3.3. Analysis of Differential Expression Gene

**Figure 3:**
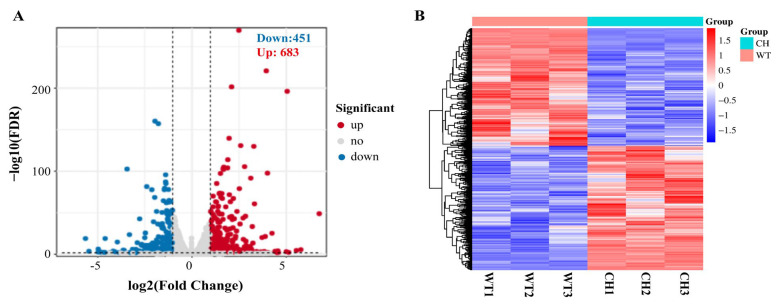
Identification of the DEGs in ribs between WT and CH groups. (**A**) Volcano plot of DEGs. Red dots indicate the up-regulated genes and blue dots indicate the down-regulated genes. (**B**) Heatmap representation of the DEGs clustering analysis Genes are arranged in a horizontal orientation, with each column representing a distinct sample. The colour red is employed to signify genes that are expressed at a high level, while the colour blue is utilised to indicate genes that are expressed at a low level. WT: wild-type silver carp; CH: P_0_ chimeras silver carp.

Considering the observed characteristics of the ribs, we conducted an investigation into the varying levels of mRNA expression between the ribs of wild-type silver carp and P_0_ chimeras. A total of 934 genes exhibiting differential expression were identified in ribs between wild-type silver carp and P_0_ chimeras, with 483 genes being up-regulated and 451 genes being down-regulated, utilising |log2 (fold change)| > 1 and FDR < 0.05 as the established significance criterion in comparison to the wild-type silver carp (Figure 3A, Table S2). Figure 3B displays the heatmap illustrating the clustering analysis of differentially expressed genes.

### 3.4. Functional Analysis of DEGs

**Figure 4:**
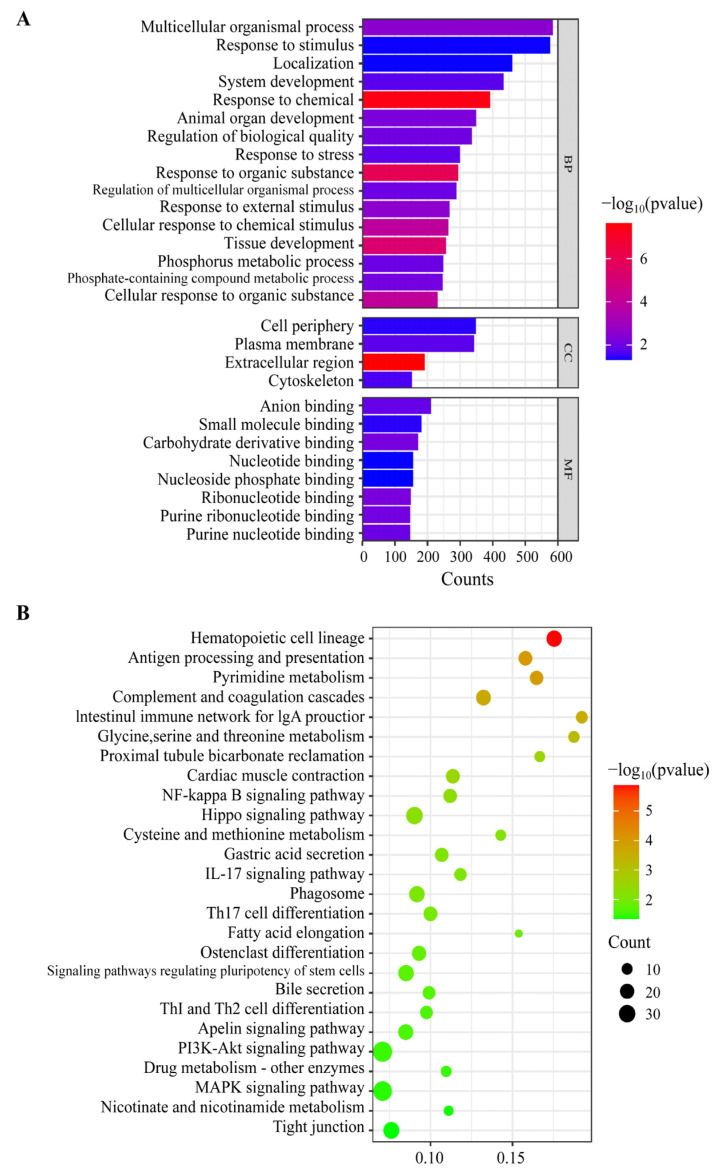
Functional enrichment analysis of the DEGs in ribs between WT and CH groups. (**A**) GO enrichment analysis of DEGs The X axis represents the number of DEGs; each bar in Y axis represents one enriched term and is coloured by −log10 (*p*-value). (**B**) KEGG enrichment analysis of DEGs. The X-axis indicates the enrichment factor, and the Y-axis indicates the KEGG pathways. The larger the bubble, the more genes enriched in the pathway, and the colour of the bubble represents the *p*-value.

To further understand the effect of bmp6 deletion on silver carp ribs, we performed a GO enrichment of DEGs. The histogram illustrates the GO terms that are highly enriched, encompassing significant biological processes (BPs), cellular components (CCs), and molecular functions (MFs). For the cellular components category, the major subcategories are “Cell periphery” (GO:0071944), “Plasma membrane” (GO:0005886), and “Extracellular region” (GO:0005576). For the molecular function category, the major subcategories are “Anion binding” (GO:0043168), “Small molecule binding” (GO:0036094), and “Carbohydrate derivative binding” (GO:0097367). For the biological processes category, “Multicellular organismal process” (GO:0032501) is the most prominently represented GO term, followed by “Response to organic substance” (GO:0010033), and “Response to chemical” (GO:0042221) (Figure 4A, Table S3).

A total of 26 KEGG signalling pathways were significantly enriched, which are shown in the bubble diagram (Figure 4B). Among these pathways, “haematopoietic cell lineage” was the most prominent. “Hippo signalling pathway”, “osteoclast differentiation”, and “NF-kappa B signalling pathway” were related to bone development. “Antigen processing and presentation”, “complement and coagulation cascades”, and “intestinal immune network for IgA production” were immune-related signalling pathways. In addition, metabolic-related signalling pathways included “pyrimidine metabolism”, “glycine, serine and threonine metabolism”, “cysteine and methionine metabolism”, and “nicotinate and nicotinamide metabolism”. “PI3K-Akt signalling pathway” and “MAPK signalling pathway”, which play important roles in complex cellular programmes like proliferation, differentiation, development, transformation, and apoptosis, are also enriched (Figure 4B, Table S4).

### 3.5. DEGs in Bone Development-Related Pathways

**Figure 5:**
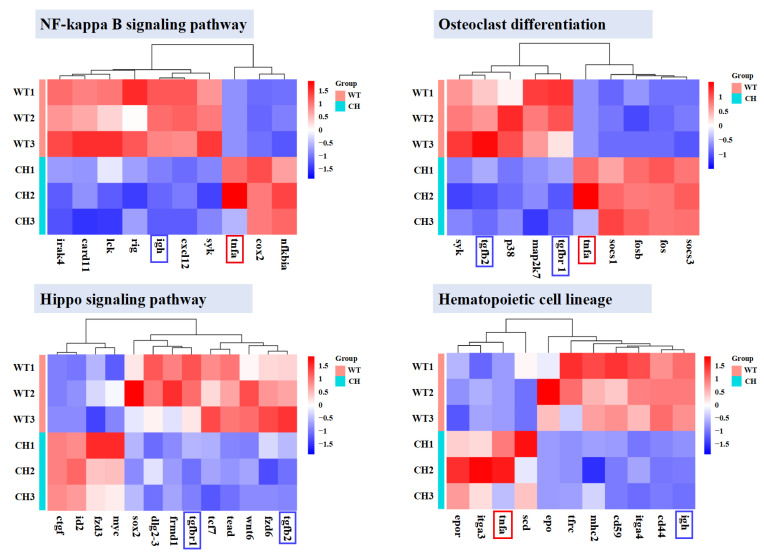
The heatmap illustrates the changes in gene expression within four pathways related to bone development. The average RPKM value of each gene is used to plot the heatmaps. Up-regulated genes are visually represented in red, whereas down-regulated genes are visually represented in blue. The genes marked with boxes are identified as participating in multiple pathways. WT: wild-type silver carp; CH: P_0_ chimeras silver carp.

Four KEGG pathways associated with bone development, including “NF-kappa B signalling pathway”, “Hippo signalling pathway”, “osteoclast differentiation” and “haematopoietic cell lineage”, have been visually depicted through heatmaps to illustrate the inclination towards differentially expressed genes within these pathways (Figure 5). The comprehension of these alterations in the DEGs of these pathways will facilitate the comprehension of the impact of *bmp6* deletion on the development of ribs in silver carp.

### 3.6. Validation of RNA-Seq Data Using qRT-PCR

**Figure 6:**
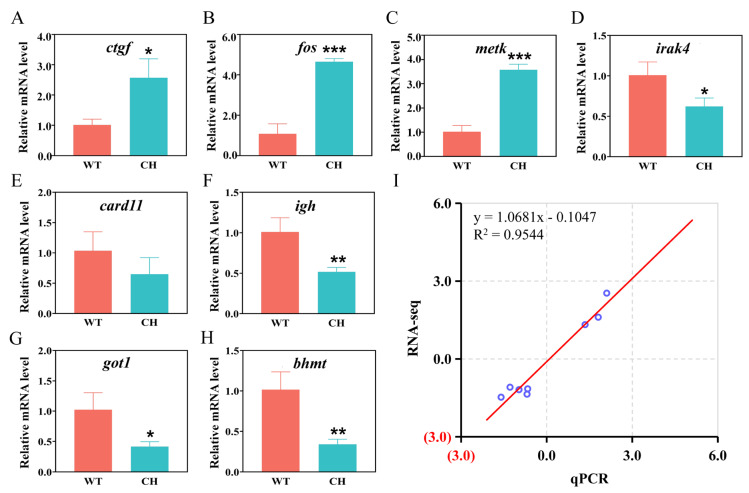
RT-qPCR validation of eight DEGs, including *ctgf* (**A**), *fos* (**B**), *metk* (**C**), *irak4* (**D**), *card1l* (**E**), *igh* (**F**), *got1* (**G**), and *bhmt* (**H**). Y-axis exhibits the relative expression level of genes. WT: wild-type silver carp; CH: P_0_ chimeras silver carp. (**I**) A correlation analysis between the RNA-seq and qRT-PCR data. The reference line indicates the linear relationship between the results of RNA-seq and qRT-PCR. Error bars represent the standard deviation of three replicates. * *p* < 0.05, ** *p* < 0.01, and *** *p* < 0.001 indicate significant differences.

To validate the accuracy of RNA-seq, eight DEGs were selected for qRT-PCR. The findings depicted in Figure 6 demonstrate a significant correlation between the outcomes obtained from qRT-PCR analysis and RNA-seq. The investigated DEGs exhibited regulation in a consistent direction (either up-regulated or down-regulated) when assessed using both techniques, thereby affirming the dependability and precision of the RNA sequencing and subsequent analysis (Table 2).

## 4. Discussion

In the present study, CRISPR/Cas9 technology was employed to repress the expression of *bmp6* on the initial exon in silver carp. Given the typical four-year maturation cycle of silver carp, we are only able to obtain the chimeras in the P_0_ generation. To guarantee the effectiveness of the suppression, three distinct targets were carefully selected (Figure 1), resulting in a remarkably efficient rate of 86.11% in the P_0_ generation (Table 1). However, conventional single-target knockouts typically demonstrate a knockout efficiency that is below 50% [25], suggesting that the implementation of multiple targets resulted in a noteworthy enhancement of knockout efficiency.

As a constituent of the BMP family, BMP6 plays a crucial role in regulating various biological processes, including bone development [26], iron metabolism [27], and female fertility [28,29]. Our current understanding supports the role of *bmp6* in the developmental processes of intermuscular bones, whereby the inhibition of *bmp6* results in a decrease or total absence of intermuscular bones in some cyprinid fish species, while not affecting growth, muscle, and bone development [7,30,31,32,33]. The results of our study revealed a comparable occurrence in silver carp. The chimeras in the P_0_ generation are fully viable and appeared normal, with approximately 70% displaying partial loss of IBs (Table 1, Figure 2). 

Additionally, Gui et al. observed that the suppression of the *Runx2b* gene led to a complete absence of IBs, with approximately half of the IB-free individuals exhibiting subtle abnormalities in rib structure, including bifurcation and warping [16]. The underlying factors contributing to the production of distinct rib phenotype remain unknown. They postulated that the deficiency of *runx2b* could potentially impact the functionality of certain genes implicated in rib development. As a key gene affecting bone development, similarly to *runx2b*, the role of *bmp6* on rib development in silver carp was first reported in this study. We observed approximately 30% of the P_0_ chimeras displaying subtle rib bifurcation or bending (Table 1, Figure 2). Nevertheless, the proportion of rib defects in fish and the severity of these defects were found to be comparatively lower in comparison to mutants exhibiting runx2b deletion. One potential explanation is that our acquisition was limited to chimeras, and the strain of *runx2b^-/-^* is homozygous. Another possible reason is that the loss of *runx2b* has a greater effect on rib development than does the loss of *bmp6.*

The utilisation of Illumina technology for transcriptome sequencing of cDNAs has emerged as a highly efficient approach for generating extensive sequences that accurately represent expressed genes [34,35,36]. Currently, transcriptomics is being effectively applied in scientific investigations concerning intermuscular bones in crucian carp, zebrafish, and carp [37,38]. Therefore, in light of the phenotypic observations, we conducted transcriptome analyses on chimeras and wild-type silver carp ribs to elucidate the impact of *bmp6* on rib development. We found that “haematopoietic cell lineage” was the most prominent, followed by the KEGG signalling pathway enriched by 934 differentially expressed genes detected, which is associated with the “Hippo signalling pathway”, “osteoblast differentiation”, and “NF signalling pathway”, which may be related to bone development (Table S2). The tendency toward the DEGs in these pathways is represented in heatmaps, and several DEGs have been identified to participate in multiple pathways (Figure 5).

Bone undergoes continuous remodelling throughout an individual’s lifespan, and any disruption in this process can lead to the development of bone defects. Two major bone cells are involved in the bone remodelling process. Osteoblasts play a crucial role in the process of bone formation, whereas osteoclasts are the cells primarily responsible for bone resorption. The integrity and function of bone are upheld through a delicate equilibrium between osteoblasts and osteoclasts [39,40].

Many studies have demonstrated that the activation of NF-κB signalling pathway by osteoclast precursors through RANKL stimulation is crucial for osteoclast differentiation [41,42,43]. TNF-α, a cytokine synthesised by activated macrophages, exerts an inhibitory effect on osteoblasts while simultaneously stimulating osteoclasts, and is considered to act by directly increasing RANK expression in macrophages and by increasing RANKL in stromal cells [44]. In the present study, the expression of *tnfα* was found to be increased in ribs of *bmp6*-knocked out chimeras, thereby suggesting a rise in osteoclasts within the ribs, consequently resulting in rib defects. In addition, the “haematopoietic cell lineage” exhibited the highest level of enrichment in the comparison with the chimeras and wild-type silver carp. TNF-α has been identified as a regulator of various signalling pathways in haematopoietic cells [45]. Macrophages, which serve as osteoclast precursor cells, originate from haematopoietic stem cells (HSCs). Therefore, aside from its role in osteoclast differentiation, TNF-α may regulate the formation of osteoclasts by regulating the differentiation of haematopoietic stem cells. Furthermore, “antigen processing and presentation” emerges as the second most prominent enrichment pathway subsequent to “haematopoietic cell lineage” (Figure 4, Figure S1). TNF-α could regulate antigen-presenting function through two mechanisms: the depletion of immunogenic peptides and the impairment of antigen-independent T cell clustering [46]. T cells, which secrete RANKL, promote osteoclastogenesis during inflammation. Our results suggest that TNF-α may play a role in inflammatory response by regulating antigen processing and presentation processes, and further regulates osteoclast generation.

The transforming growth factor-beta (TGF-β) superfamily encompasses more than forty members, including TGF-βs, Nodal, Activin, and bone morphogenetic proteins (BMPs) [47]. The signalling pathways of TGF-β/BMPs are widely acknowledged for their involvement in bone formation during mammalian development and demonstrate diverse regulatory functions within the organism [48]. TGF-β signalling promotes osteoprogenitor proliferation, early differentiation, and commitment to the osteoblastic lineage. Mice with a deficiency in TGF-β1 exhibit diminished bone growth and mineralization [49], while mice with a double knockout of TGF-β2 and TGF-β3 demonstrate an absence of distal portions of the rib [50]. The receptors of TGF-β isoforms, including type I receptor (Tgfbr1) and type II receptor (Tgfbr2), also play important roles in endochondral and intramembranous ossification. Mice with tissue-specific deletion of Tgfbr1 through Dermo1-Cre exhibit shortened and widened long bones, diminished bone collars, and decreased trabecular bone density [51]. The elimination of Tgfbr2 through Col2a1-Cre in mice leads to various abnormalities in the cranial base and vertebrae [52]. The elimination of Tgfbr2 driven by Prx-Cre yields deficiencies in the long bones, joints [53], and skull vault [54], thereby demonstrating the indispensable involvement of Tgfbr2 in both intramembranous and endochondral bone formation. The findings of our investigation demonstrate that the proliferation and differentiation of osteoprogenitor cells may be impeded in the ribs of *bmp6*-knockout chimeras through reducing the expression levels of *tgfb2* and *tgfbr1*, ultimately resulting in the manifestation of rib defects.

The Fos gene family consists of four members: *fos*, *fosb*, *fosl1*, and *fosl2*. In our study, the expression levels of *fos* and *fosb* were found to be elevated in the ribs of *bmp6*-knockout chimeras, thereby exerting a detrimental impact on rib development. The findings of previous studies align with our results, showing that both the overexpression and deletion of the *fos* have detrimental impacts on bone development [55]. Connective tissue growth factor (*CTGF*) belongs to the CCN family and contains cysteine-rich stromal cell protein, plays a crucial role in regulating numerous biological events, including development, wound healing, and cardiogenesis [56,57]. Previous data provide confirmation that *CTGF* serves as a distinctive growth factor, facilitating the proliferation and differentiation of osteoclast [57,58,59]. In this study, *ctgf* involved in *“*Hippo signalling pathway” was up-regulated in *bmp6*-knockout chimeras, indicating that the osteoclastogenesis was enhanced.

Overall, our transcriptome analysis in silver carp ribs revealed that the disruption of bmp6 results in up-regulation of genes such as *tnfα*, *fos*, and *ctgf*, as well as the down-regulation of genes such as *tgfb2* and *tgfbr1*. This molecular alteration may promote the proliferation and differentiation of osteoclasts while hindering the formation and specialisation of osteoblasts, ultimately culminating in the manifestation of rib defects. However, further investigation is warranted to explore various aspects of this subject, such as the heritability of rib defects and the underlying mechanisms responsible for such abnormalities. The chimeras exhibiting rib defects in the P_0_ generation will undergo proliferation, allowing for the detailed observation of rib development. If the heritability of the rib defect is stable, an analysis of the expression patterns in offspring will be conducted to further elucidate the underlying cause of the observed phenotype.

## 5. Conclusions

In the present study, CRISPR/Cas9 technology was employed to repress the expression of bmp6 on the initial exon in silver carp with a remarkably efficient rate of 86.11% in the P_0_ generation. The chimeras are fully viable and appeared normal, with approximately 70% displaying partial loss of IBs, and approximately 30% displaying rib bifurcation or bending. A transcriptome analysis on chimeras and wild-type silver carp ribs was conducted, resulting in the detection of 483 up-regulated genes and 451 down-regulated genes. A KEGG analysis showed that four bone development-related pathways, including “NF-kappa B signalling pathway”, “Hippo signalling pathway”, “osteoclast differentiation”, and “haematopoietic cell lineage”, were significantly enriched. The up-regulation of *tnfα*, *fos*, and *ctgf* genes may facilitate the proliferation and differentiation of osteoclasts, whereas the down-regulation of *tgfb2* and *tgfbr1* genes may hinder the formation and specialisation of osteoblasts, ultimately resulting in rib abnormalities. These findings provide insights into the function of bmp6 in the context of rib development in silver carp, while simultaneously providing theoretical support for the cultivation of high-quality IB-free fish.

## Figures and Tables

**Table 1:** The phenotypic statistics of the chimeras in the P_0_ generation.

Classification	Wild-Type Silver Carp	P_0_ Chimeras
*Bmp6* knockout efficiency	-	86.11%
Survival percentage	79.2%	77.4%
Normal physical appearance	100%	100%
Fish exhibiting diminished IBs	0/15	13/18
The mean quantity of Ibs	113	74
The reduction ratio of Ibs	-	34.5%
Fish exhibiting rib bifurcation	0/15	3/18
Fish exhibiting rib bending	0/15	6/18

**Table 2:** Comparisons of RNA-seq and RT-qPCR results.

Gene Abbreviation	Gene Description	Fold Change (WT vs. CH)	
		qPCR	RNA-Seq
*ctgf*	Connective tissue growth factor	2.54	2.50
*fos*	Proto-oncogene	4.30	5.80
*metk*	S-adenosyl methionine synthase isoform	3.50	3.05
*igh*	Ig mu chain C region membrane-bound	0.51	0.44
*irak4*	Interleukin-1 receptor-associated kinase	0.62	0.39
*got1*	Aspartate aminotransferase, cytoplasmic	0.41	0.47
*bhmt*	Betaine—homocysteine S-methyltransferase 1	0.33	0.36
*card11*	Caspase recruitment domain-containing protein 11	0.63	0.45

## Data Availability

The data presented in this study are available in the article.

## Supplementary Materials

The following supporting information can be downloaded at: https://www.mdpi.com/article/10.3390/ani14101451/s1, Figure S1: The heatmap illustrates the changes in gene expression within the “antigen processing and presentation” pathway; Table S1: Primers used in this study; Table S2: Genes exhibiting differential expression in ribs between wild type and P_0_ chimeras; Table S3: GO terms enriched by DEGs in ribs between WT and CH groups; Table S4: KEGG pathways enriched by DEGs in ribs between WT and CH groups.
