# Supplementary material for: Transcriptome Analysis Elucidates the Potential Key Genes Involved in Rib Development in bmp6-Deficient Silver Carp (Hypophthalmichthys molitrix)
Source: Animals (Basel). 2024 May 13;14(10):1451. doi: 10.3390/ani14101451 (PMC11117292; doi:10.3390/ani14101451)
Supplement: Supplementary file 1 [file animals-14-01451-s001.zip › Table S1.pdf]

Table S1. Primers used in this study.

Primer name	Sequences (5'−3')
bmp6-gRNA1-F	TAATACGACTCACTATAGGAAGAGATTCTGTCCATACGTTTTAGAGCTAGAAATAGC
bmp6-gRNA2-F	TAATACGACTCACTATAGGCCCAACTTCATACATCGGGTTTTAGAGCTAGAAATAGC
bmp6-gRNA3-F	TAATACGACTCACTATAGGCAGGTAGTAGTTCTGTACGTTTTAGAGCTAGAAATAGC
sgRNA Scaffold Primer	AAAAGCACCGACTCGGTGCCACTTTTTCAAGTTGATAACGGACTAGCCTTATTTTAACTTGCTATTTCTAGCTCTAAAAC
bmp6- mutant screening-F	CACCCTCATCATCATCACCAC
bmp6- mutant screening-R	CAGCATGAAGAGGGGTGCTG
fos-qPCR-F	ACTGGGAGCCACTCTACACA
fos-qPCR-R	AGTGAGGAGGGTTGGGGAAT
bhmt-qPCR-F	GAGAGGATACGTGAAGGCCG
bhmt-qPCR-R	TGCATGACATTTGACCCGGA
card11-qPCR-F	GCACGATACCGAGCTACTCC
card11-qPCR-E	TAGCTCTCGACAGACGTGGA
ctgf-qPCR-F	ATGGGTAGTGGCTCAGGAGT
ctgf-qPCR-R	TCGCAAACATCTCGCTCTGT
metk-qPCR-F	CCTCTCGTGCTGTAGTGGAC
metk-qPCR-R	CAAGGTGAACCCCTTGAGCA
igh-qPCR-F	GTACGCTGATGTGCAATGCT
igh-qPCR-R	TTCTGCGTGTTCAACTGTGC
irak4-qPCR-F	GCCCAACGGATCTCTACTGG
irak4-qPCR-R	CGTCAAACCGAAGTCGGAGA
got1-qPCR-F	TTAGGCCTGCCAGAGTTTCG
got1-qPCR-R	TTCCATTGTACCAACGGCGA
