# Supplementary figures and images for: Transcriptome Analysis Elucidates the Potential Key Genes Involved in Rib Development in bmp6-Deficient Silver Carp (Hypophthalmichthys molitrix)
Source: Animals (Basel). 2024 May 13;14(10):1451. doi: 10.3390/ani14101451 (PMC11117292; doi:10.3390/ani14101451)

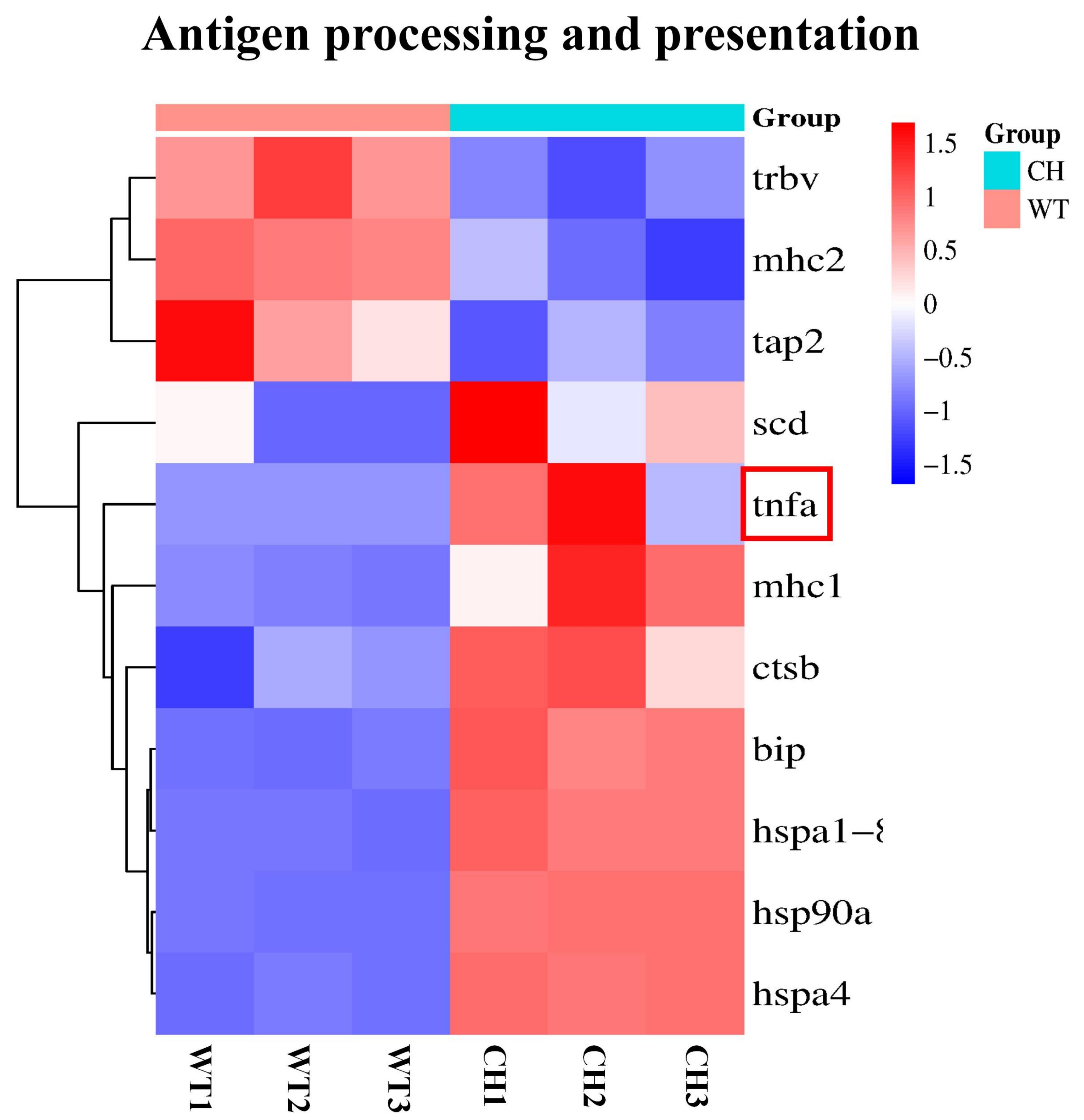

Supplement: Supplementary file 1 [file animals-14-01451-s001.zip › Figure S1.jpg]
